# G**α**_11_ mutation in mice causes hypocalcemia rectifiable by calcilytic therapy

**DOI:** 10.1172/jci.insight.91103

**Published:** 2017-02-09

**Authors:** Caroline M. Gorvin, Fadil M. Hannan, Sarah A. Howles, Valerie N. Babinsky, Sian E. Piret, Angela Rogers, Andrew J. Freidin, Michelle Stewart, Anju Paudyal, Tertius A. Hough, M. Andrew Nesbit, Sara Wells, Tonia L. Vincent, Stephen D.M. Brown, Roger D. Cox, Rajesh V. Thakker

**Affiliations:** 1 Academic Endocrine Unit, Radcliffe Department of Medicine, University of Oxford, Oxford, United Kingdom.; 2 Department of Musculoskeletal Biology, Institute of Ageing and Chronic Disease, University of Liverpool, Liverpool, United Kingdom.; 3 ARUK Centre for Osteoarthritis Pathogenesis, The Kennedy Institute of Rheumatology, University of Oxford, Oxford, United Kingdom.; 4 Medical Research Council (MRC) Mammalian Genetics Unit and Mary Lyon Centre, MRC Harwell Institute, Harwell Science and Innovation Campus, United Kingdom.; 5 Biomedical Sciences Research Institute, Ulster University, Coleraine, United Kingdom.

## Abstract

Heterozygous germline gain-of-function mutations of G-protein subunit α_11_ (Gα_11_), a signaling partner for the calcium-sensing receptor (CaSR), result in autosomal dominant hypocalcemia type 2 (ADH2). ADH2 may cause symptomatic hypocalcemia with low circulating parathyroid hormone (PTH) concentrations. Effective therapies for ADH2 are currently not available, and a mouse model for ADH2 would help in assessment of potential therapies. We hypothesized that a previously reported dark skin mouse mutant (*Dsk7*) — which has a germline hypermorphic Gα_11_ mutation, Ile62Val — may be a model for ADH2 and allow evaluation of calcilytics, which are CaSR negative allosteric modulators, as a targeted therapy for this disorder. Mutant *Dsk7/+* and *Dsk7/Dsk7* mice were shown to have hypocalcemia and reduced plasma PTH concentrations, similar to ADH2 patients. In vitro studies showed the mutant Val62 Gα_11_ to upregulate CaSR-mediated intracellular calcium and MAPK signaling, consistent with a gain of function. Treatment with NPS-2143, a calcilytic compound, normalized these signaling responses. In vivo, NPS-2143 induced a rapid and marked rise in plasma PTH and calcium concentrations in *Dsk7/Dsk7* and *Dsk7/+* mice, which became normocalcemic. Thus, these studies have established *Dsk7* mice, which harbor a germline gain-of-function Gα_11_ mutation, as a model for ADH2 and have demonstrated calcilytics as a potential targeted therapy.

## Abstract 2

A mouse with a germline gain-of-function Gα11 mutation provides a model for autosomal dominant hypocalcemia type 2 (ADH2), which is rectifiable by calcilytic therapy.

## Introduction

Autosomal dominant hypocalcemia (ADH) is a disorder of systemic calcium homeostasis caused by increased sensitivity of the calcium-sensing receptor (CaSR) signaling pathway to extracellular calcium (Ca^2+^_o_) concentrations (1, 2). The CaSR is a widely expressed class C G-protein coupled receptor (GPCR) that plays a pivotal role in Ca^2+^_o_ homeostasis by transducing elevations in the prevailing Ca^2+^_o_ concentration into multiple signaling cascades that include G_q/11_-protein–mediated activation of phospholipase C (PLC), which in the parathyroid glands induce increases in intracellular calcium (Ca^2+^_i_) and MAPK signaling responses that lead to decreased parathyroid hormone (PTH) secretion (3). ADH is associated with hypocalcemia, hyperphosphatemia, hypomagnesemia, and inappropriately low or normal PTH concentrations; some patients may also be hypercalciuric (1, 4) or develop a Bartter-like syndrome characterized by hypokalemic alkalosis, renal salt wasting, and hyperreninemic hyperaldosteronism (5, 6). Approximately 50% of patients develop symptomatic hypocalcemia, and >35% have ectopic calcifications within the kidneys and basal ganglia (1, 2, 7, 8). ADH is a genetically heterogeneous disorder comprised of two variants, which are known as ADH type 1 (ADH1) and ADH type 2 (ADH2). ADH1 (OMIM #601198) is caused by germline mutations of the CaSR, which is encoded by the *CASR* gene on chromosome 3q21.1 (1, 9), whereas, ADH2 (OMIM #615361) is caused by germline mutations of the widely expressed G-protein subunit α_11_ (Gα_11_) protein, which is encoded by the *GNA11* gene on chromosome 19p13.3 (2, 7, 8, 10). Both CaSR and Gα_11_ mutations identified in ADH patients have been demonstrated to enhance CaSR-mediated Ca^2+^_i_ and MAPK signaling in cellular studies, consistent with a gain of function (1, 2, 7, 9–11).

Mouse models with gain-of-function *Casr* mutations have been generated by chemical mutagenesis and knock-in strategies, and they have been shown to have a phenotype closely resembling ADH1 in humans with hypocalcemia, hyperphosphatemia, reduced PTH concentrations, hypercalciuria, and ectopic calcifications (12, 13). Such models have been utilized to evaluate calcilytic compounds, which are negative allosteric CaSR modulators that represent a potential targeted therapy for ADH (14–17). Indeed, a long-acting amino-alcohol calcilytic compound known as NPS-2143 has been shown in vivo to rectify the hypocalcemia of *Nuf* mice, which harbor a germline gain-of-function *Casr* mutation, Leu723Gln (13, 18). Recent in vitro studies have also revealed NPS-2143 to normalize the gain of function caused by Gα_11_ mutations that lead to ADH2 (11). However, it remains unclear whether this calcilytic may rectify the hypocalcemia associated with ADH2, and mouse models that harbor *Gna11* mutations in association with hypocalcemia have not been reported to be available for such in vivo studies. Such mouse models would also aid the further phenotypic characterization of ADH2, as limited information is available from the small numbers of patients studied to date (2, 7, 8, 10, 19).

To develop a mouse model for ADH2, we conducted studies to investigate the calcitropic phenotype of a previously reported mouse mutant known as *Gna11^Mhdadsk7^* or “dark skin 7”, and henceforth referred to as *Dsk7*, which has increased dermal pigmentation in association with a germline hypermorphic *Gna11* mutation, Ile62Val (formerly referred to as Ile63Val) (20). We hypothesized that *Dsk7* mice would exhibit dysregulation of Ca^2+^_o_ homeostasis in keeping with ADH2. Our study has revealed *Dsk7* mice to have hypocalcemia and reduced PTH concentrations, which are caused by a gain-of-function *Gna11* mutation that leads to upregulation of CaSR-mediated Ca^2+^_i_ and MAPK signaling responses. Moreover, we demonstrate that NPS-2143 treatment rectifies this gain of function in vitro and ameliorates the hypocalcemia of *Dsk7* mice.

## Results

### Genotype and phenotype studies of Dsk7 mice.

DNA sequence analysis of *Gna11* in *Dsk7* mice confirmed the reported A-to-G transition (c.184A>G, RefSeq Accession NM_002067.4) at codon 62 of the Gα_11_ protein, resulting in an Ile to Val missense substitution (Supplemental Figure 1, A and B; supplemental material available online with this article; https://doi.org/10.1172/jci.insight.91103DS1) (20). This mutation also led to the loss of a *FokI* restriction endonuclease site (Supplemental Figure 1B), as reported (20), which was used to confirm the presence of the mutation in *Dsk7* mice (Supplemental Figure 1, C and D) and for genotyping of subsequent generations. Analysis of offspring bred from crosses of *Dsk7/+* × *Dsk7/+* mice showed that the proportion bred as homozygous-affected *Dsk7/Dsk7* mice were >30% less (*x^2^* = 10.20, degrees of freedom [df] = 2, *P* < 0.01) than would be expected from a Mendelian pattern of inheritance (Supplemental Table 1). The *Dsk7/Dsk7* mice also had significantly reduced body weight and increased skin pigmentation (data not shown), when compared with age-matched WT (*+/+*) mice (Table 1), as previously reported (20).

**Figure 1:**
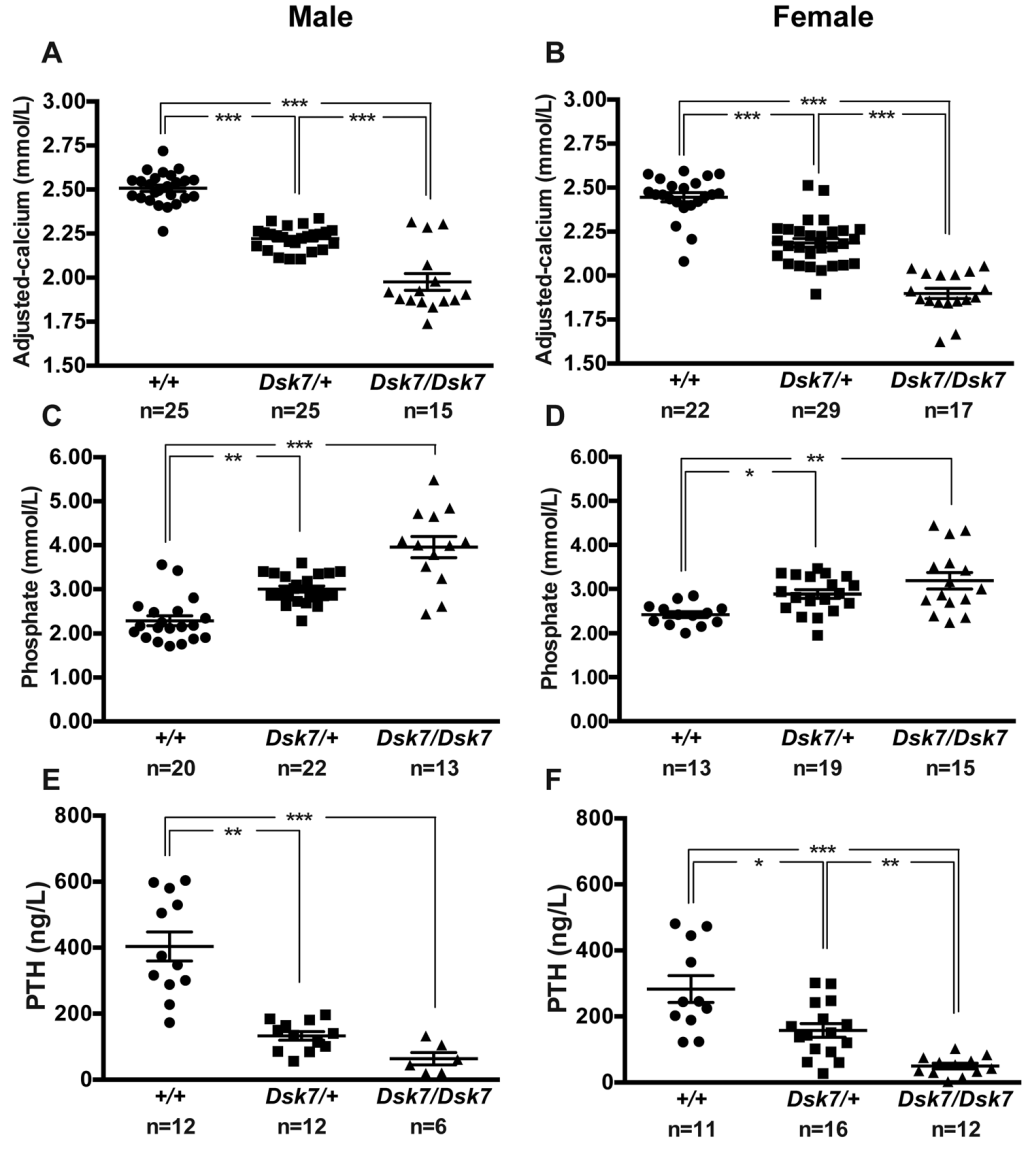
Calcitropic phenotype of *Dsk7* mice. (**A** and **B**) Plasma adjusted-calcium, (**C** and **D**) plasma phosphate, and (**E** and **F**) plasma PTH concentrations of male and female WT (+/+, circles), *Dsk7/+* (squares), and *Dsk7/Dsk7* (triangles) mice, respectively. Mean ± SEM values for the respective groups are indicated by the solid bars. *P < 0.05, **P < 0·01, ***P < 0.001. A Kruskal-Wallis test followed by Dunn’s test for nonparametric pairwise multiple comparisons were used for analysis of **A**–**F**.

Biochemical analysis of plasma samples collected under isoflurane terminal anesthesia revealed male and female *Dsk7/+* and *Dsk7/Dsk7* mice to be significantly hypocalcemic and hyperphosphatemic, and to have significantly reduced PTH concentrations when compared with *+/+* mice (Figure 1 and Table 1). The hypocalcemia was significantly more marked in *Dsk7/Dsk7* mice compared with heterozygous-affected *Dsk7/+* mice (Figure 1 and Table 1). Reduced FGF-23 concentrations were noted in *Dsk7/Dsk7* mice compared with *+/+* mice (Table 1). No significant differences were observed in plasma urea and creatinine concentrations, alkaline phosphatase activity, or 1,25-dihydroxyvitamin D concentrations (Table 1). Twenty-four–hour urinary calcium excretion and urinary calcium/creatinine ratios were significantly reduced in female *Dsk/+* mice, although fractional excretion of calcium was not different between either male or female *Dsk7* mice and respective *+/+* mice (Table 2). The tubular maximum reabsorption of phosphate (TmP) was significantly increased in *Dsk7/+* and *Dsk7/Dsk7* mice (Table 2), consistent with the low circulating PTH concentrations of these mice (Figure 1 and Table 1) (21). Fractional excretion of sodium and potassium were not altered (Table 2). Whole body dual-energy X-ray absorptiometry (DXA) showed no differences in bone mineral density (BMD) between male and female *Dsk7* mice and respective *+/+* mice (Table 3). μCT analysis of trabecular bone also showed no alterations in the bone volume fraction or in the thickness or number of trabeculae between male or female *Dsk7* mice and respective *+/+* mice (Table 3). Ophthalmological examination did not reveal any lens opacifications in *Dsk7/Dsk7* mice (data not shown), and these findings contrast with *Nuf* mice, which harbor a gain-of-function *Casr* mutation in association with hypocalcemia and cataracts (18).

### Effects of Ile62Val Gα_11_ mutation on Gα_11_ structure and CaSR-mediated signaling.

**Figure 2:**
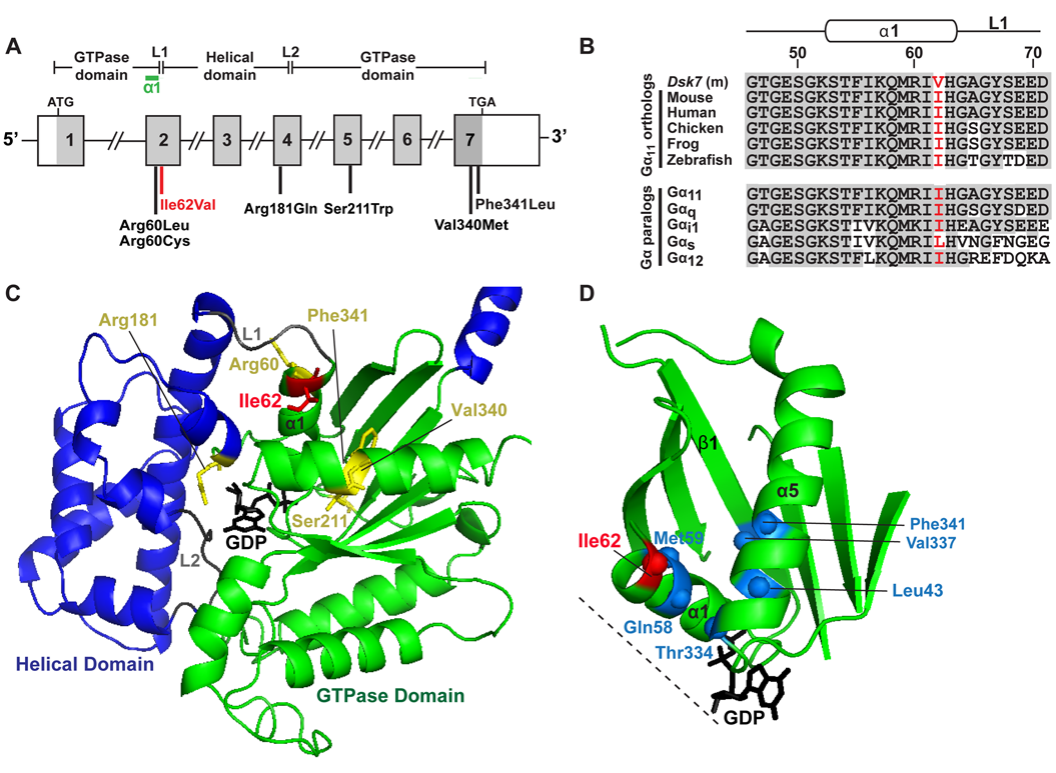
Structural characterization of the Ile62Val Gα_11_ mutation. (**A**) Genomic organization of *Gna11* showing location of the Ile62Val mutation. The Gα_11_ GTPase domain (encoded by exon 1, 5′portion of exon 2, 3′ portion of exon 4 and exons 5–7) is connected to the helical domain (encoded by the 3′ portion of exon 2, exon 3, and 5′ portion of exon 4) by the linker 1 (L1) and 2 (L2) peptides. The Ile62Val mutation (red) lies within the α1 helix (green). The location of reported ADH2 mutations are indicated (black). (**B**) Multiple protein sequence alignment of residues comprising the α1 helix and L1 peptide of Gα_11_-subunit orthologs (top) and Gα-subunit paralogs (bottom). Conserved residues are shown in gray. The WT (Ile) and Dsk7 mutant (m) (Val) residues are shown in red. (**C**) Homology model of the Gα_11_ protein. The Gα helical (blue) and GTPase (green) domains are connected by the L1 and L2 peptides (gray). GDP (black) is bound at the interdomain interface. Previously reported residues mutated in ADH2 are shown in yellow. The mutated Ile62 residue is shown in red. (**D**) Close-up view of the Ile62 residue, which lies within a hydrophobic cluster of residues (blue spheres) on α1, α5, and β1 of the GTPase domain and near to the interdomain interface (dotted line) and GDP binding site.

The Ile62 residue is located within the α1 helix of the GTPase domain of Gα_11_ and close to the Arg60 residue, which has been reported to be mutated in ADH2 patients (Figure 2A) (7, 8). The WT Ile62 residue, which is encoded in exon 2 of the *Gna11* gene (Figure 2A), is absolutely conserved in Gα_11_ orthologs and highly conserved in Gα-subunit paralogs (Figure 2B). To determine the importance of the WT Ile62 residue for Gα_11_ function, homology modeling was undertaken using the crystal structure of the related Gα_q_ and Gα_i_ proteins (22, 23). This revealed the WT Ile62 residue to be located at the interface between the Gα-subunit helical and GTPase domains (Figure 2C) and to comprise part of a conserved hydrophobic cluster of amino acid residues within the GTPase domain of the Gα-subunit, which play a key role in stabilizing G-proteins in an inactive GDP-bound conformation (Figure 2D) (23). These findings predicted that mutation of the conserved WT Ile62 Gα_11_ residue to a mutant Val62 residue would destabilize the inactive GDP-bound Gα-subunit, thereby leading to guanine-nucleotide exchange and Gα_11_ activation (23).

**Figure 3:**
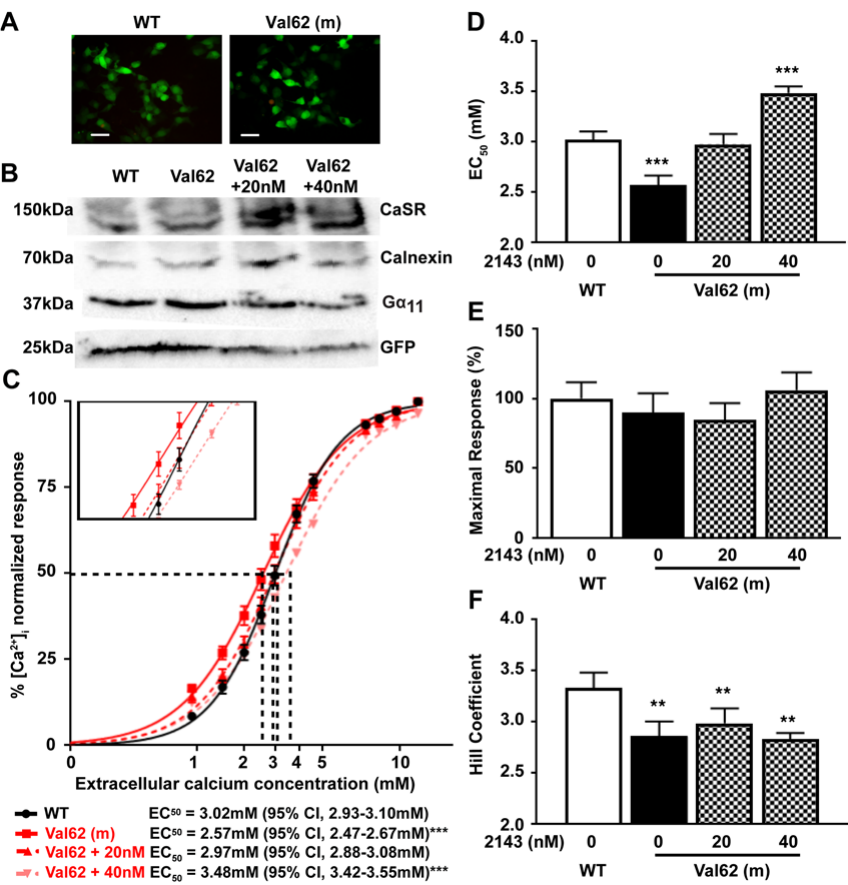
Intracellular calcium responses of the Val62 Gα_11_ mutant and effect of NPS-2143 treatment. (**A**) Fluorescence microscopy of HEK293 cells stably expressing CaSR (HEK-CaSR) and transiently transfected with WT Ile62 or mutant (m) Val62 pBI-CMV2-*GNA11* constructs. GFP expression in these cells indicates successful transfection and expression by these constructs. Scale bars: 10 μm. (**B**) Western blot analysis of lysates from HEK-CaSR cells used for flow cytometry experiments. Transient transfection with WT or mutant Val62 expression constructs resulted in overexpression of Gα_11_ and GFP. Calnexin, a housekeeping protein, and untransfected cells (Supplemental Figure 2) were used as controls. (**C**) Ca^2+^_i_ response to changes in [Ca^2+^]_o_ of HEK-CaSR cells transfected with WT or Val62 Gα_11_ mutant. The Ca^2+^_i_ responses to changes in [Ca^2+^]_o_ are expressed as a percentage of the maximum normalized responses and shown as the mean ± SEM of 5–8 assays from 2 independent transfections. The Val62 Gα_11_ mutant led to a leftward shift in the concentration-response curve (red line). The addition of 20 nM NPS-2143 rectified the leftward shift of the Val62 Gα_11_ mutant (red dashed line), whereas 40 nM NPS-2143 led to a rightward shift of the mutant concentration-response curve (pink dashed line) compared with WT (black line). (**D**–**F**) Histograms showing mean ± SEM EC_50_ values, % maximal signaling responses, and Hill coefficients, respectively, for cells expressing WT (open bar) or Val62 Gα_11_ mutant (black bar) proteins and for mutant-expressing cells treated with NPS-2143 (patterned bars). **P < 0.01, ***P <0.001. F-test for **C**–**D**. Mann-Whitney *U* test for **E**–**F**.

To determine the effects of these predicted changes in Gα_11_ structure on CaSR-mediated signaling, HEK293 cells stably expressing the CaSR (HEK-CaSR) were transiently transfected with pBI-CMV2-*GNA11* constructs expressing either the WT (Ile62) or mutant (Val62) Gα_11_ proteins. This bidirectional pBI-CMV2 vector allows for coexpression of Gα_11_ and GFP at equivalent levels (2); expression of the CaSR, Gα_11_, and GFP was confirmed by fluorescence microscopy and/or Western blot analyses (Figure 3, A and B). The expression of Gα_11_ was shown to be similar in cells transiently transfected with WT or mutant proteins and shown to be greater than that observed in untransfected cells (Supplemental Figure 2). The expression of mutant Gα_11_ in transfected cells, together with that of the endogenous expression of WT Gα_11_, corresponds to the heterozygous situation in vivo (Figure 3B and Supplemental Figure 2). The responses of Ca^2+^_i_ to alterations in [Ca^2+^]_o_ of cells expressing the different *GNA11* vectors were assessed by flow cytometry. The Ca^2+^_i_ responses in WT and mutant Gα_11_-expressing cells were shown to increase in a dose-dependent manner following stimulation with increasing concentrations of Ca^2+^_o_. However, responses in mutant Val62-expressing cells were significantly elevated compared with WT-expressing cells (Figure 3C). Thus, the Val62 mutant–expressing cells showed a leftward shift in the concentration-response curve (Figure 3C), with significantly reduced mean half-maximal response (EC_50_) value (*P* < 0.001, *n* = 7) of 2.57 mM (95% CI, 2.47–2.67 mM) for Val62-expressing cells, compared with 3.02 mM (95% CI, 2.93–3.10 mM) for WT-expressing cells (Figure 3D), consistent with a gain of function of the Gα_11_ mutant, as observed with Gα_11_ mutations that lead to ADH2 (2, 7, 10, 11). The Val62 Gα_11_ mutant did not alter the maximal Ca^2+^_i_ signaling responses (Figure 3E) but was associated with a significant reduction of the Hill coefficient (*P* < 0.01) (Figure 3F).

### In vitro effects of NPS-2143 treatment on Ca^2+^_i_ and MAPK responses of the Val62 Gα_11_ mutant.

To investigate whether allosteric inhibition of the CaSR can rectify the gain of function associated with the Ile62Val Gα_11_ mutation, NPS-2143 was added at 20 and 40 nM concentrations, as similar doses of this calcilytic have been reported to rectify the altered signaling responses associated with ADH2-causing Gα_11_ mutations (11). An assessment of Ca^2+^_i_ responses showed 20 nM NPS-2143 to increase the EC_50_ of Val62-expressing mutant cells to 2.97 mM (95% CI, 2.88–3.08 mM), so that this was not significantly different from the EC_50_ of untreated WT cells (Figure 3, C and D). However, addition of NPS-2143 at the higher 40 nM dose increased the EC_50_ of mutant cells to a value of 3.48 mM (95% CI, 3.42–3.55 mM), which was significantly greater than that of untreated WT cells (*P* < 0.001) (Figure 3, C and D). NPS-2143 had no effect on the maximal responses or Hill coefficients of mutant-expressing cells (Figure 3, E and F).

**Figure 4:**
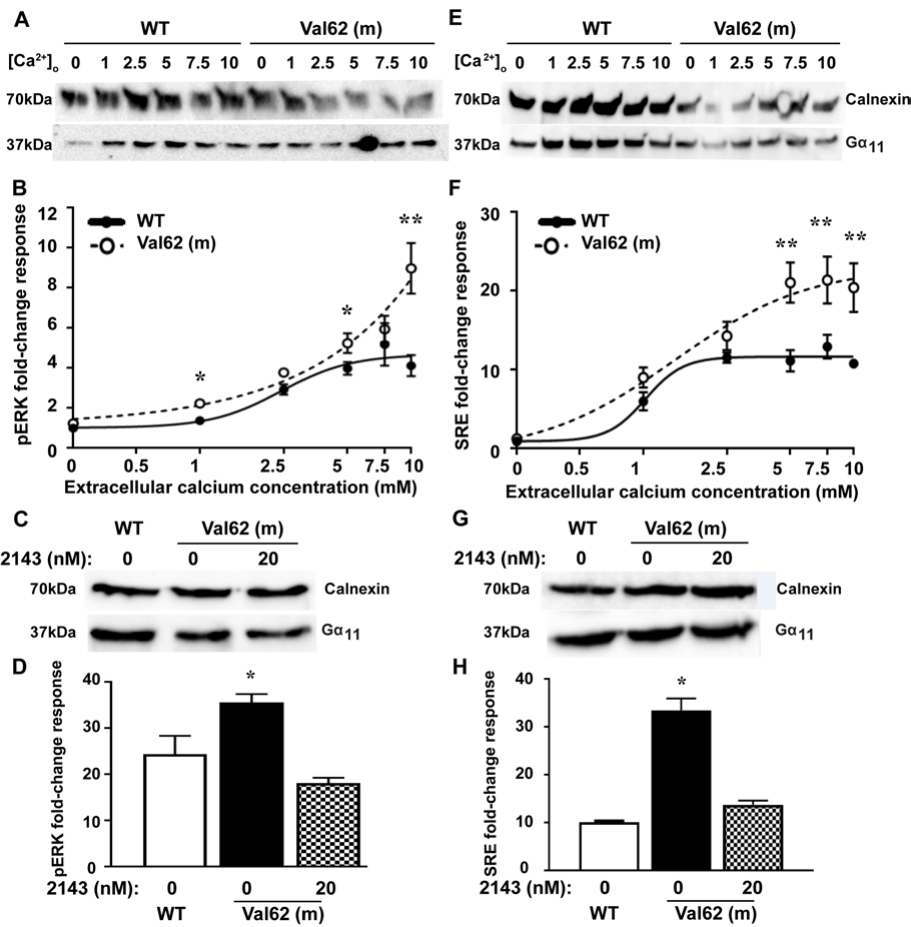
MAPK responses of the Val62 Gα_11_ mutant and effect of NPS-2143 treatment. (**A**) Western blot analysis of lysates from HEK-CaSR cells used for phosphorylated ERK (pERK) experiments. (**B**) pERK fold-change responses to changes in [Ca^2+^]_o_ of cells transfected with WT (solid line) or Val62 Gα_11_ mutant (dashed line). (**C**) Western blot analysis of lysates from cells used to assess effect of NPS-2143 (2143) on pERK responses. (**D**) Effect of NPS-2143 on pERK responses of Val62 Gα_11_ mutant. (**E**) Western blot analysis of lysates from HEK-CaSR cells used for serum response element (SRE) reporter experiments. (**F**) SRE reporter fold-change responses to changes in [Ca^2+^]_o_ of cells transfected with WT (solid line) or Val62 Gα_11_ mutant (dashed line). (**G**) Western blot analysis of lysates from cells used to assess effect of NPS-2143 on SRE reporter responses. (**H**) Effect of NPS-2143 on the SRE reporter responses of the Val62 Gα_11_ mutant. The Val62 Gα_11_ mutant led to significantly increased pERK and SRE fold-change responses following stimulation with Ca^2+^_o_. In the absence of Ca^2+^_o_, the pERK and SRE responses of the Val62 Gα_11_ mutant were not significantly different from WT, thereby indicating the Ile62Val Gα_11_ mutation to be nonconstitutively activating. The addition of 20 nM NPS-2143 decreased the pERK and SRE responses of cells expressing the Val62 Gα_11_ mutant (patterned bars) compared with untreated cells (solid bars), so that these were not significantly different from WT (open bars). The fold-change responses are shown as the mean ± SEM of 4–8 independent transfections. *P < 0.05, **P < 0.01. Mann-Whitney *U* test for **B**, **D**, **F**, and **H**.

Some gain-of-function Gα_11_ mutations have been reported to promote tumorigenesis by constitutively upregulating MAPK signaling (24), and we therefore assessed the oncogenic potential of the Ile62Val Gα_11_ mutation in HEK-CaSR cells by measurements of phosphorylated ERK (pERK), which is a key component of the MAPK cascade (7, 25). WT and mutant pBI-CMV2-*GNA11* vectors were transiently transfected into HEK-CaSR cells, and the fold-change increases in pERK proteins were measured following exposure to varying [Ca^2+^]_o_. Western blot analysis confirmed expression of WT and mutant Gα_11_ proteins in cells used for the pERK experiments (Figure 4A). In the absence of Ca^2+^_o_ stimulation, the basal pERK responses of the Val62 mutant were not significantly different from cells expressing WT Gα_11_ (Figure 4B), whereas exposure to Ca^2+^_o_ led to significantly increased pERK fold-change responses of cells expressing the Val62 Gα_11_ mutant (Val62 = 9.4 ± 1.4) when compared with cells expressing WT Gα_11_ (Ile62 = 4.1 ± 0.5, *P* < 0.01) (Figure 4B). NPS-2143 was next added to cells expressing the Val62 Gα_11_ mutant protein (Figure 4C) and which had been stimulated with 10 mM Ca^2+^_o_. Exposure to 20 nM of this calcilytic compound normalized the elevated pERK responses (Figure 4D). The effect of the Val62 Gα_11_ mutant on MAPK signaling was also investigated by measuring gene transcription induced by a serum-response element (SRE) containing luciferase reporter construct, which is a downstream mediator of ERK signaling (7, 25). Western blot analysis confirmed expression of WT and mutant Gα_11_ proteins in cells used for the SRE reporter experiments (Figure 4E). In the absence of Ca^2+^_o_ stimulation, the basal SRE reporter activity of the Val62 mutant was not significantly different from cells expressing WT Gα_11_ (Figure 4F), whereas Ca^2+^_o_ stimulation led to significantly increased SRE reporter fold-change responses of cells expressing the Val62 Gα_11_ mutant (Val62 = 20.7 ± 1.5 compared with 11.0 ± 0.4 for the Ile62 WT Gα_11_, *P* < 0.01) (Figure 4F). NPS-2143 was then added at a dose of 20 nM to cells expressing the Val62 Gα_11_ mutant protein (Figure 4G), in the presence of 10 mM Ca^2+^_o_, and this normalized the increased SRE reporter responses (Figure 4H).

### In vivo effects of NPS-2143 on the hypocalcemia of Dsk7 mice.

**Figure 5:**
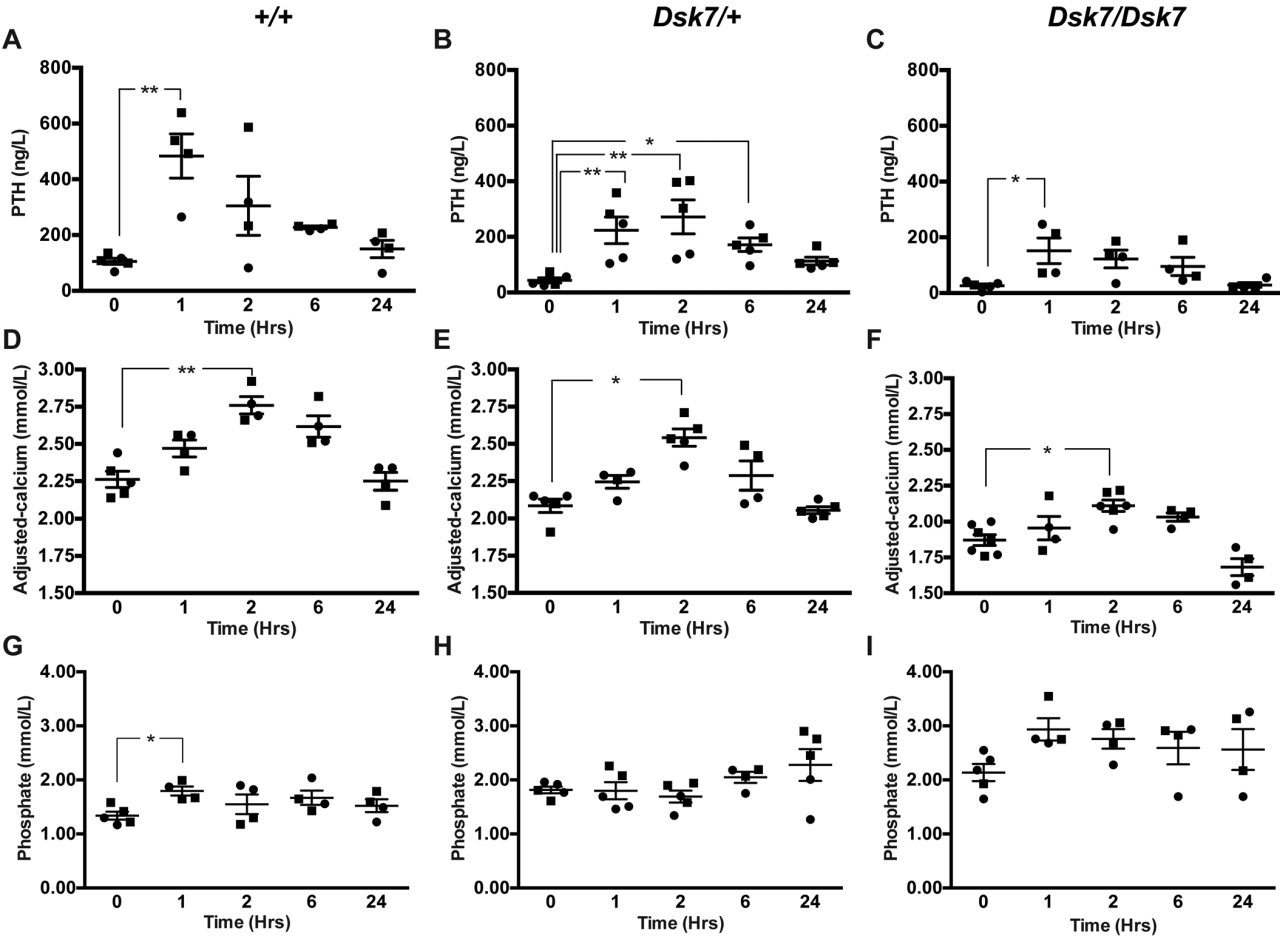
Effect of NPS-2143 on plasma PTH, calcium, and phosphate of *Dsk7* mice at 0, 1, 2, 6, and 24 hours after dose. (**A–C**) Plasma PTH, (**D–F**) plasma adjusted-calcium, and (**G–I**) plasma phosphate concentrations of WT (+/+), *Dsk7/+*, and *Dsk7/Dsk7* mice, respectively. Mean values for the respective groups are indicated by solid bars. *n* = 4–7 mice per study time point. Squares, males; circles, females. *P < 0.05, **P < 0.01. A Kruskal-Wallis test followed by Dunn’s test for nonparametric pairwise multiple comparisons were used for analysis of **A**–**I**.

We next assessed whether the hypocalcemia of *Dsk7* mice may be improved by treatment with NPS-2143. Previous studies have demonstrated that i.p. administration of a 30 mg/kg NPS-2143 dose significantly increases plasma calcium and PTH concentrations in *Nuf* mice with a gain-of-function *Casr* mutation, but it is associated with adverse effects on renal function (13). In contrast, a study using oral gavage administration of NPS-2143 in WT rats has reported that a dose of 100 μmol/kg can increase PTH responses without adverse renal effects (26). We therefore evaluated the effects of i.p. and oral gavage administration of NPS-2143 in *+/+* mice, and we showed that a single oral gavage 100 μmol/kg dose of NPS-2143 significantly increased plasma calcium without altering renal function, whereas i.p. injection of this calcilytic led to a significant rise in plasma urea and creatinine (Supplemental Figure 3). We therefore administered a 100 μmol/kg bolus of NPS-2143 by oral gavage to *+/+*, *Dsk7/+*, and *Dsk7/Dsk7* mice and measured PTH, calcium, phosphate, urea, and creatinine concentrations at 0, 1, 2, 6, and 24 hours after dose using plasma samples obtained from the lateral tail vein following application of topical local anesthesia. Administration of NPS-2143 led to a 4- to 5-fold elevation in plasma PTH concentrations in *+/+*, *Dsk7/+*, and *Dsk7/Dsk7* mice, with a maximal rise in PTH occurring at 1–2 hours after dose (Figure 5, A–C). The rise in PTH was associated with a significant increase in plasma calcium concentrations in *+/+*, *Dsk7/+,* and *Dsk7/Dsk7* mice, which were maximally elevated (0.25–0.5 mmol/l above baseline values) at 2 hours after dose (Figure 5, D–F). Indeed, administration of 100 μmol/kg NPS-2143 normalized the plasma calcium concentrations of *Dsk7/Dsk7* mice, whereas *Dsk7/+* mice became transiently hypercalcemic (Figure 5, D–F). NPS-2143 treatment also led to a transient rise in plasma phosphate concentrations in *+/+* and *Dsk7/Dsk7* mice (Figure 5, G–I), which was not associated with any increases in plasma urea or creatinine (Supplemental Figure 4). Thus, these studies demonstrate that a single dose of NPS-2143 can rectify the hypocalcemia of *Dsk7/+* and *Dsk7/Dsk7* mice.

## Discussion

Our studies have demonstrated that *Dsk7* mice, which harbor a germline Ile62Val *Gna11* mutation (20), are hypocalcemic, are hyperphosphatemic, and have reduced circulating PTH concentrations. Thus, *Dsk7* mice represent a mouse model for the human disorder of ADH2, which is caused by gain-of-function *GNA11* mutations (2, 7, 8, 10). In support of this, the Ile62Val Gα_11_ mutant protein enhanced the signaling responses of CaSR-expressing cells in vitro, and this data indicates that the *Gna11* mutation is leading to the observed phenotype in *Dsk7* mice. The Ile62Val *Gna11* mutation showed a gene dosage effect with the heterozygous-affected *Dsk7/+* mice having an ~0.3 mmol/l decrease in plasma calcium and ~65% reduction in PTH concentrations compared with +/+ mice, whereas homozygous-affected *Dsk7/Dsk7* mice have significantly more pronounced hypocalcemia (plasma calcium ~0.5 mmol/l lower than +/+ mice) and a greater (~85%) reduction in PTH concentrations (Figure 1 and Table 1). The more severe hypocalcemia of homozygous-affected mice may potentially have affected their viability, thereby providing an explanation for the observed reduced numbers of mice with this genotype. The degree of hypocalcemia of affected *Dsk7* mice is similar to that of ADH2 patients, who have been reported to have serum calcium values ranging between 0.1–0.6 mmol/l below that of unaffected family members (7, 8, 27). Moreover, *Dsk7* mice generally had detectable circulating PTH concentrations and also a normal fractional excretion of calcium, which is in keeping with the majority of ADH2 patients (2, 7, 8). The markedly reduced circulating PTH of *Dsk7/Dsk7* mice was not significantly associated with decreases in plasma 1,25-dihydroxyvitamin D concentrations (Table 1), and such effects of PTH may have been counteracted by the low plasma FGF-23 concentrations, which would be expected to increase 1,25-dihydroxyvitamin D (28). Serum FGF-23 concentrations have not been reported for ADH2 patients, and our finding of low FGF-23 values in *Dsk7/Dsk7* mice was most likely a consequence of the hypocalcemia, which has been previously shown to reduce circulating FGF-23 concentrations in parathyroidectomized rats and in rats with hypocalcemia due to dietary calcium restriction (29). However, the mechanism by which hypocalcemia lowers FGF-23 remains to be established (29). The bone phenotype of ADH2 has been evaluated in a single kindred by plain radiography, and no abnormalities were identified (19). Our skeletal assessment of *Dsk7* mice also did not reveal any major alterations in BMD or trabecular volume or structure, which indicates that gain-of function Gα_11_ mutations may not lead to alterations in bone metabolism.

In addition to *Dsk7* mice having similar calcitropic features to ADH2 patients, *Dsk7* mice may also share some noncalcitropic phenotypes with affected patients. Indeed, short stature has been reported in 2 ADH2 kindreds (7, 19), and in keeping with this, *Dsk7/Dsk7* mice have been previously noted to have significantly reduced body length and weight compared with +/+ mice (20). However, a key species-specific difference is that *Dsk7/+* and *Dsk7/Dsk7* mice have increased skin pigmentation, due to an expansion of the melanocyte population within the dermis (30), and such alterations in skin color have not been reported for ADH2 patients to date. The studies of *Dsk7* mice and ADH2 patients have also revealed differences to the phenotype of ADH1, which is caused by gain-of-function *CASR* mutations (1, 9). In particular, ADH1 patients and knock-in mice harboring ADH1-causing *Casr* mutations have been demonstrated to have a relative or absolute hypercalciuria and high bone mass (1, 12), in addition to hypocalcemia and low circulating PTH concentrations. These alterations in parathyroid, renal, and bone metabolism observed in ADH1 are in keeping with the known expression and function of the CaSR in these tissues (31–33). In contrast, our finding that *Dsk7* mice have hypocalcemia and low PTH values in the absence of renal or bone abnormalities suggests that germline Gα_11_ mutations may influence CaSR signaling responses in the parathyroid gland without perturbing the function of this GPCR in other calcitropic tissues (Figure 1 and Tables 1–3). In support of this, studies of mice with a parathyroid-specific ablation of the Gα_q/11_ proteins (34) have demonstrated a critical role for these proteins in PTH secretion, whereas it remains to be established if these G-proteins act as a signaling partner for the CaSR in the kidneys and bone. Moreover, the lack of a renal or bone phenotype in *Dsk7* mice may also have been caused by alterations in the relative expression of Gα_11_ and Gα_q_. Thus, if Gα_q_ expression predominates in kidney and bone, then a gain of function of Gα_11_ may have been insufficient to alter CaSR signaling responses in these tissues. An additional difference in the phenotypes associated with gain-of-function mutations of the CaSR and Gα_11_ proteins is that *Nuf* mice — which have hypocalcemia in association with a germline *Casr* mutation, Leu723Gln — developed cataracts by 4–6 weeks of age (18), whereas *Dsk7* mice do not have lens abnormalities. The CaSR has been reported to be expressed and functionally linked to Ca^2+^-activated K^+^ channels in lens epithelial cells (35); however, its role in cataract formation has not been determined. The absence of lens opacifications in *Dsk7* mice indicates that alterations in CaSR-mediated Gα_11_ signaling may not be involved in the development of this eye disorder.

Our findings help to elucidate some of the mechanisms whereby the germline Ile62Val Gα_11_ mutation, which involves the substitution of amino acid residues with similar hydrophobic properties, causes a gain of function and enhances the sensitivity of CaSR-expressing cells to Ca^2+^_o_. The WT Gα_11_ Ile62 residue appears to be absolutely conserved among multicellular organisms and is located within the Gα-subunit α1 helix at the interdomain interface, which contains a hydrophobic cluster of residues involved in guanine nucleotide binding (Figure 2) (23). Upon binding of G-protein to GPCR, the α1 helix has been shown to undergo conformational changes that destabilize the interdomain interface, thereby leading to the exchange of GDP for GTP (23), which triggers G-protein activation. It is likely that the Ile62Val mutation leads to a gain of function by decreasing the stability of the α1 helix, and this possibility is supported by reported mutagenesis studies involving the related Gα_i_ protein, which have shown substitution of the homologous Gα_i_ Ile residue with alanine to destabilize the GDP-bound form of the Gα-subunit (23). The Gα_11_ interdomain interface and hydrophobic residue cluster represent a hotspot for gain-of-function mutations, with 5 out of 6 of the reported ADH2-causing mutations situated within this region (2, 7, 8, 10), consistent with our proposal that these mutations likely alter the conformation of the guanine nucleotide-binding pocket, thereby promoting GDP-GTP exchange and G-protein activation.

Although the Val62 Gα_11_ mutant protein enhanced the Ca^2+^_i_ responses of CaSR-expressing cells, consistent with a gain of function, it also significantly reduced the Hill coefficient, which was not rectifiable with calcilytic treatment (Figure 3). The Hill coefficient is commonly used to represent cooperative receptor-ligand interactions (36) but may also indicate the degree of interaction between a GPCR and its downstream partner signaling protein (25). Our finding of a reduced Hill coefficient suggests that the Ile62Val mutation may have altered coupling of the mutant Gα_11_ protein with the CaSR. In support of this, ADH1-causing mutations located within the CaSR transmembrane domain, which is predicted to be the site of G-protein binding, have also been reported to lower the Hill coefficient, while leading to a gain of function (37).

The most severe clinical manifestation of *GNA11* mutations is uveal melanoma, which is a malignant tumor arising from the melanocytes of the choroid plexus, ciliary body, and iris and which is caused by somatic mutations of the Arg183 and Qln209 Gα_11_ residues that lead to constitutive upregulation of MAPK signaling (24). As the Ile62Val *Gna11* mutation is associated with increased proliferation of melanocyte precursors in *Dsk7* mice (20), we assessed the oncogenic potential of this mutation in vitro and demonstrated that it increased MAPK responses only in the presence of Ca^2+^_o_ stimulation (Figure 4). Thus, the Ile62Val Gα_11_ mutant does not harbor constitutive activity, and these findings are consistent with previously reported ADH2 mutations, which also lead to a nonconstitutive gain of function (7, 11) and are supported by studies of double mouse mutants, which have shown that the Ile62Val *Gna11* mutation does not cause increased skin pigmentation in the absence of an upstream functional receptor (20).

ADH2 is frequently associated with hypocalcemic symptoms such as paraesthesia, muscle cramps, carpo-pedal spasm, and seizures (2, 7, 8, 10). However, treatment of symptomatic patients with calcium and active vitamin D preparations has resulted in adverse effects such as hypercalciuria, nephrocalcinosis, and nephrolithiasis (7, 10). Although calcilytic compounds represented targeted therapies for patients with CaSR mutations causing symptomatic forms of ADH1 (12, 13), it was unclear if these CaSR allosteric modulators may rectify abnormalities of the downstream Gα_11_ protein, and thus have potential benefit for ADH2 patients. Recent in vitro studies have revealed that low doses (10 nM to 30 nM concentrations) of the NPS-2143 calcilytic compound can successfully correct the gain of function associated with ADH2-causing Gα_11_ mutations (11). Consistent with these findings, a similar dose of NPS-2143 also normalized the Ca^2+^_i_ and MAPK responses of the Ile62Val Gα_11_ mutant (Figures 3 and 4). Moreover, we have shown that administration of a single NPS-2143 dose induced a rapid and marked rise in PTH concentrations in *Dsk7/+* and *Dsk7/Dsk7* mice, and rectified or improved their hypocalcemic phenotypes, respectively (Figure 5); these data suggest that *Dsk7/Dsk7* mice will likely require a higher dose of NPS-2143 to correct their hypocalcaemia. These results suggest that calcilytics such as NPS-2143 will likely be of benefit for ADH2 patients, who also harbor heterozygous *GNA11* mutations (2, 7, 8, 10). Oral administration of NPS-2143 was well tolerated, and the acute rise in plasma calcium after dose did not affect the health or condition of the mice, nor did it influence biochemical parameters such as electrolytes or renal function. However, an increase in plasma phosphate was noted in WT mice following treatment with NPS-2143, and this finding has also been reported in *Nuf* mice, which became hyperphosphatemic and had increases in urea and creatinine following a single i.p. injection of NPS-2143 (13). However, the rise in phosphate observed in the present study was not associated with alterations in renal function, and the cause of the hyperphosphatemia remains to be elucidated. Longer-term studies involving repetitive dosing are required to confirm the efficacy and safety of calcilytics for improving circulating calcium concentrations in the setting of ADH2. Of note, blood samples obtained from mice under isoflurane terminal anesthesia (Table 1) showed significant elevations in plasma concentrations of phosphate and PTH compared with samples obtained using topical local anesthesia (Supplemental Figure 5). General anesthetics, which include inhalation agents such as isoflurane, have been reported in laboratory animal studies to significantly alter plasma concentrations of phosphate and PTH (38, 39). Our findings further highlight the effects of anesthetic agents on these commonly measured biochemical parameters.

In conclusion, we have established that *Dsk7* mice, which harbor a germline gain-of-function Gα_11_ mutation, represent an in vivo model for the human disorder of ADH2. We have also utilized this model to demonstrate that calcilytic compounds have the potential to treat the hypocalcemia associated with this disorder.

## Methods

### Animals.

*Dsk7* mice were rederived on a C3H strain background (C3H.C3HeB/FeJ-Gna11^Mhdadsk7^/IegH) by in vitro fertilization using sperm obtained from the European Mutant Mouse Archive (EMMA). All study mice were aged between 12–16 weeks and housed in a controlled environment at the MRC Harwell Institute in accordance with UK Home Office and MRC welfare guidance. Mice had free access to water and were fed ad libitum on a commercial diet (RM3, Special Diet Services) that contained 1.15% calcium, 0.58% phosphate, and 4089 IU/kg of vitamin D.

### Compounds.

NPS-2143 hydrochloride (also known as 2-Chloro-6-[(2R)-3-[[1,1-dimethyl-2-(2-naphthalenyl)ethyl]amino]-2-hydroxypropoxy]-benzonitrile hydrochloride) was obtained from Sigma-Aldrich (catalog SML0362) and dissolved in a 20% aqueous solution of 2-hydroxypropyl-β-cyclodextrin (Sigma-Aldrich, catalog H107) prior to use in in vitro and in vivo studies.

### DNA sequence analysis.

Genomic DNA was extracted from auricular biopsies, as described (40), and gene-specific primers were used to perform PCR amplification and DNA sequence analysis of *Gna11* exon 2, as reported (2). The germline *Gna11* mutation was confirmed by Fok*I* restriction endonuclease analysis (New England Biolabs), as previously described (20).

### Protein sequence alignment and 3-dimensional modeling.

Protein sequences of Gα_11_ were aligned using ClustalOmega (http://www.ebi.ac.uk/Tools/msa/clustalo/) (41). PyMOL Molecular Graphics System (Version 1.2r3pre, Schrödinger, PyMOL) (16) was used to model the effects of the Gα_11_ Ile62Val mutation. Gα_11_ 3-dimensional (3-D) modeling was undertaken using the reported 3-D structure of Gα_q_ in complex with the small molecule inhibitor YM-254890 (Protein Data Bank accession no. 3AH8) (22) and also using the reported structure of the Gα_i_ protein (Protein Data Bank accession no. 1GDD) (23).

### Cell culture and protein expression.

Functional studies were undertaken using a human *GNA11* construct (2), as the human and mouse Gα_11_ proteins share an overall amino acid identity of 98% and are 100% identical in the region surrounding the mutated site. The Val62 mutation was introduced by site-directed mutagenesis (QuikChange Lightning, Agilent Technologies) into a pBI-CMV2-*GNA11* expression construct, as described (2). WT and mutant pBI-CMV2-*GNA11* constructs were transiently transfected into HEK293 cells that stably express the full-length human *CASR* cDNA (HEK-CaSR), as described (2, 42, 43). HEK293 cells were used because suitable parathyroid and renal tubular cells are not available, and HEK293 cells have been established as a model for the functional expression of Gα_11_ proteins (2, 43, 44). HEK-CaSR cells were cultured in high-glucose DMEM (Invitrogen) supplemented with 10% FBS and 1% geneticin at 37°C, 5% CO_2_ (2). Successful transfection was confirmed by visualizing GFP fluorescence using an Eclipse E400 fluorescence microscope with an epifluorescence filter, and images were captured using a DXM1200C digital camera and NIS Elements software (Nikon) (2, 44). The expression of Gα_11_ and CaSR proteins was confirmed by Western blot analyses using Gα_11_ (D-6, sc-390382, Santa Cruz Biotechnologies Inc.), anti-GFP (B-2, sc-9996, Santa Cruz Biotechnologies Inc.), anti-calnexin (AB2301, Millipore), anti-GAPDH (AM4300, Ambion), or anti-CaSR (5C10, ADD, ab19347, Abcam) antibodies. The Western blots were visualized using an Immuno-Star Western C kit (Bio-Rad) on a Bio-Rad Chemidoc XRS+ system (2, 42). Densitometric analysis was performed using Image J analysis software (Version 1.46; rsb.info.nih.gov/ij/), and statistical analysis was performed using 2-way ANOVA, as previously described (45).

### Measurement of Ca^2+^_i_ responses.

The effect of the mutant Gα_11_ protein on the Ca^2+^_i_ responses of CaSR-expressing cells was assessed by a flow cytometry–based assay, as reported (2, 43, 44). Briefly, 48 hours after transfection, the cells were harvested, washed in calcium- and magnesium-free HBSS (Invitrogen), and loaded with 1 μg/ml indo-1-acetoxymethylester (Indo-1-AM; Molecular Probes) for 1 hour at 37°C (2, 10, 11, 42). After the removal of free dye, the cells were resuspended in calcium- and magnesium-free HBSS and maintained at 37°C. Transfected HEK-CaSR cells were incubated with either a 20% aqueous solution of 2-hydoxypropyl-β-cyclodextrin (vehicle) or negative allosteric modulator NPS-2143 at concentrations of 20 nM and 40 nM for 1 hour, as previously described (11).

Transfected cells in suspension were then stimulated by sequentially adding calcium to increase the [Ca^2+^]_o_ in a stepwise manner from 0–15 mM and then analyzed on a MoFlo modular flow cytometer (Beckman Coulter) by simultaneous measurement of GFP expression (at 525 nm), Ca^2+^_i_-bound Indo-1AM (at 410 nm), and free Indo-1AM (at 485nm), using a JDSU Xcyte UV laser (Coherent Radiation) on each cell at each [Ca^2+^]_o_, as described (2, 42, 43). Cytomation Summit software was used to determine the peak mean fluorescence ratio of the transient response after each individual stimulus expressed as a normalized response (2, 42, 43). Concentration-response curves were generated using a 4-parameter nonlinear regression curve-fit model (GraphPad Prism) to calculate the EC_50_ and Hill coefficient values (2, 10, 11, 42, 44). The maximal signaling response was measured as a fold-change of the peak transient Ca^2+^_i_ response to the basal Ca^2+^_i_ response. The maximal signaling response of mutant-expressing cells was expressed as a percentage of the WT maximal signaling response (11).

### Measurement of ERK1/2 phosphorylation.

HEK-CaSR cells were seeded in 48-well plates and transfected with 200 ng WT or mutant Gα_11_ proteins 24 hours prior to conducting the assays. Transfected cells were incubated in serum-free media 12 hours prior to treatment of cells with 0–10 mM CaCl_2_. Cells were lysed in Surefire lysis buffer, and AlphaScreen Surefire ERK assays measuring phosphorylated and total proteins were performed as previously described (11). For studies with negative allosteric modulators, cells were incubated with either a 20% aqueous solution of 2-hydoxypropyl-β-cyclodextrin (vehicle) or NPS-2143 for 4 hours prior to being stimulated with 10 mM CaCl_2_. The fluorescence signal in both assays was measured using the PheraStar FS microplate reader (BMG Labtech) (11, 45).

### Measurement of serum response element (SRE) luciferase reporter activity.

HEK-CaSR cells were seeded in 48-well plates and transiently transfected with 100 ng/ml Gα_11_ WT or mutant proteins, 100 ng pGL4-*SRE* luciferase reporter construct, and 10 ng/ml pRL control vector for 48 hours (Promega). Cells were incubated in serum-free media for 12 hours, followed by treatment of cells for 4 hours with 0–10 mM CaCl_2_. Cells were lysed and assays performed using Dual-Glo luciferase (Promega) on a Veritas Luminometer (Promega), as previously described (25, 45).

### Plasma biochemistry and hormone analysis.

Blood samples were collected from the lateral tail vein of study mice following application of topical local anesthesia, as reported (40), or collected from the retro-orbital vein under isoflurane terminal anesthesia. Lateral tail vein sampling provides small volumes of blood that are adequate for analysis of plasma calcium, albumin, phosphate, PTH, urea, and creatinine. However, retro-orbital vein sampling under terminal general anaesthesia is required to obtain larger blood volumes that permit analysis of a wider range of biochemical parameters. Plasma was separated by centrifugation at 5,000 *g* for 10 minutes at 8°C and analyzed for sodium, potassium, total calcium, magnesium, phosphate, urea, creatinine, albumin, and alkaline phosphatase on an Beckman Coulter AU680 analyzer, as described previously (13). Plasma calcium was adjusted for variations in albumin concentrations using the formula: (plasma calcium (mmol/l) – [(plasma albumin (g/l) – 30) × 0.02], as reported (40). Hormones were measured as follows: PTH using a 2-site ELISA kit (Immunotopics); intact FGF-23 using a 2-site ELISA kit (Kainos Laboratoties); and 1,25-dihydroxyvitamin D measured by a 2-step process involving purification by immunoextraction and quantification by enzyme immunoassay (Immunodiagnostic Systems), as described (46).

### Metabolic cages and urine biochemistry analysis.

Mice were individually housed in metabolic cages (Techniplast) and fed ad libitum on water and powdered chow. Mice were allowed to acclimatize to their environment over a 72-hour period, as described (47), prior to collection of 24-hour urine samples. Urine was analyzed for sodium, potassium, creatinine, phosphate, and calcium on a Beckman Coulter AU680 analyzer, as reported (13). The fractional excretion of sodium, potassium, and calcium were calculated using the formula U_x_/P_x_ × P_Cr_/U_Cr_, where U_x_ is the urinary concentration of the filtered substance (substance *x*) in mmol/l, P_x_ is the plasma concentration of substance *x* in mmol/l, U_Cr_ is the urinary concentration of creatinine in mmol/l, and P_Cr_ is the plasma concentration of creatinine in mmol/l (13). The ratio of TmP to GFR (TmP/GFR) was calculated using the following formula: P_Pi_ × (1 – [U_Pi_/P_Pi_ × P_Cr_/U_Cr_ ]), where P_Pi_ is the plasma concentration of phosphate and U_Pi_ is the urine concentration of phosphate.

### Skeletal imaging.

BMD was assessed by whole body DXA scanning, which was performed on mice anesthetized by inhaled isoflurane and using a Lunar Piximus densitometer (GE Medical Systems), as reported (46). DXA images were analyzed using Piximus software, as reported (46). Trabecular bone volume and structure were assessed by μCT analysis of the proximal tibia using a SkyScan 1174 scanner (SkyScan), with X-ray settings 50 kV, 800 μA, 12.6 μm isometric voxel resolution, and 0.7 degree rotation step. The tibial trabecular region, located 1.5 mm distally from the growth plate, was selected for analysis, and a volume of interest was delineated by drawing within the cortex of the trabecular region. A threshold of 80–255 density units was selected to distinguish mineralized trabecular tissue from surrounding soft tissue of the marrow cavity. Cross-sectional images were obtained and 3-D reconstruction undertaken using Skyscan CT Analyzer software (version 1.9.3.0).

### In vivo administration of NPS-2143.

NPS-2143 was administered as a single 100 μmol/kg (~45 mg/kg) dose by oral gavage or as a single 30 mg/kg dose by i.p. injection, as described (13), and plasma samples were collected by tail vein bleed at either 0, 1, 2, 6, or 24 hours after dose.

### Statistics.

All in vitro studies involved between 2–12 separate transfection experiments and between 4–8 technical assays. For the in vitro measurement of Ca^2+^_i_ EC_50_ responses, statistical comparisons were undertaken using the *F*-test (2, 11), whereas, Ca^2+^_i_ maximal signaling responses and Hill coefficients were analyzed using the Mann-Whitney *U* test. Measurements of ERK phosphorylation and SRE gene luciferase reporter activity were analyzed using the Mann-Whitney *U* test or by 2-way ANOVA with Tukey’s multiple-comparisons test. For the in vivo studies, a Kruskal-Wallis test was undertaken for multiple comparisons, and any significant differences identified were further assessed using the Dunn’s test for nonparametric pairwise multiple comparisons. All analyses were undertaken using GraphPad Prism software, and a value of *P* < 0.05 was considered significant for all analyses.

### Study approval.

Animal studies were approved by the MRC Harwell Institute Ethical Review Committee and were licensed under the Animal (Scientific Procedures) Act 1986, issued by the UK Government Home Office Department (PPL30/3271).

## Author Contributions

CMG, FMH, SAH, MAN, TLV, SDMB, RDC, and RVT designed research studies; CMG, SAH, VNB, SEP, AR, AJF, MS, AP, TAH, and SW conducted experiments; CMG, FMH, SAH, VNB, and SEP acquired and analyzed data; and CMG, FMH, SAH, and RVT wrote the manuscript.

## Supplementary Material

Supplemental data

## Figures and Tables

**Table 3:**
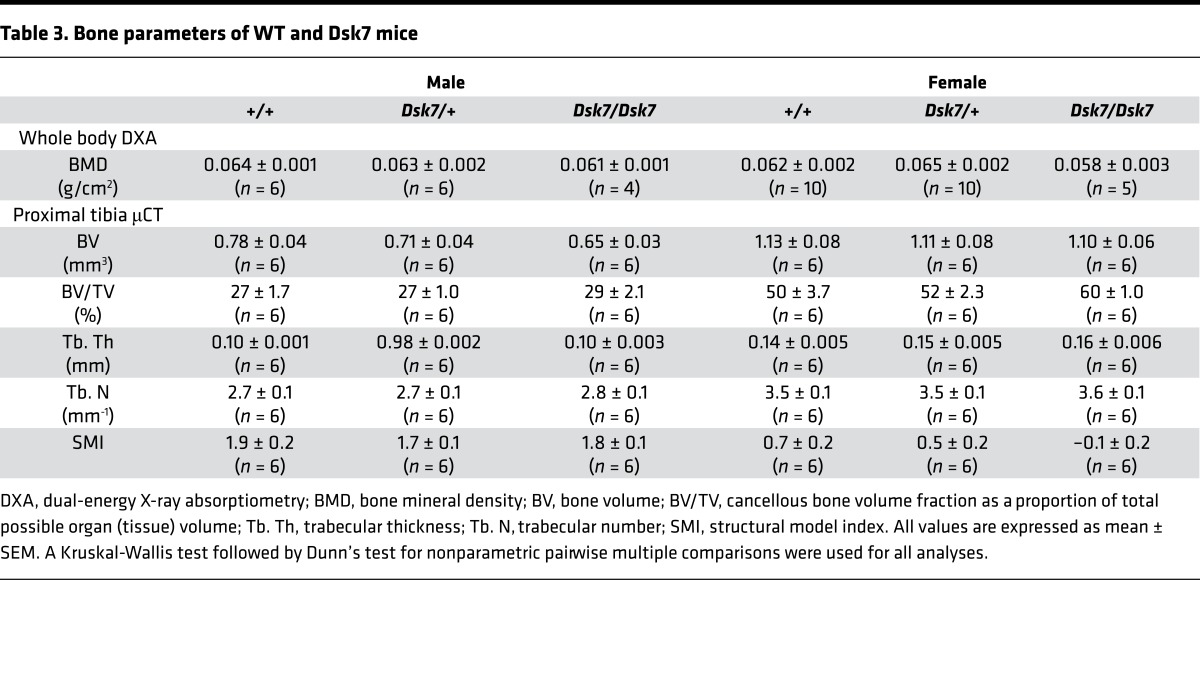
Bone parameters of WT and Dsk7 mice

**Table 2:**
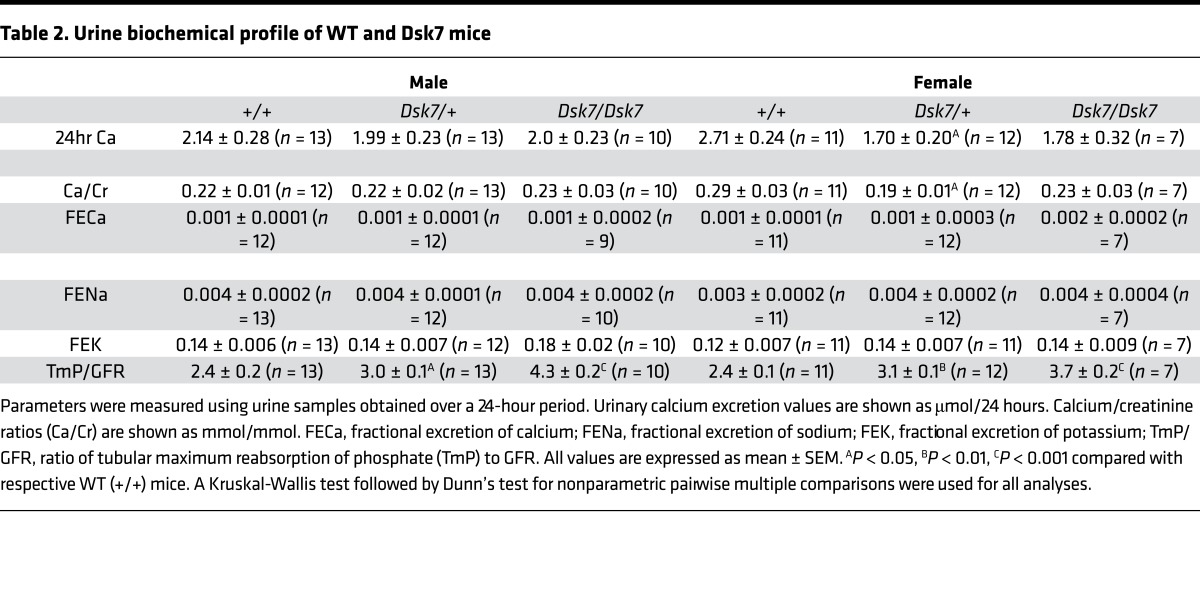
Urine biochemical profile of WT and Dsk7 mice

**Table 1:**
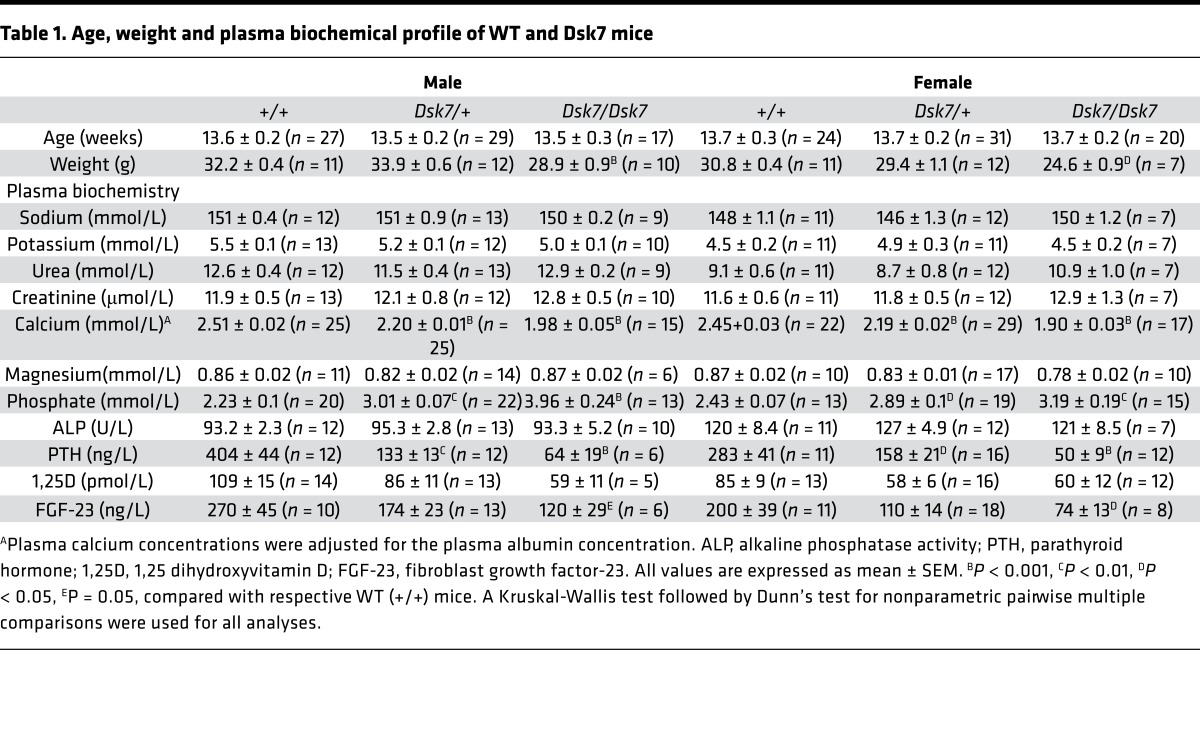
Age, weight and plasma biochemical profile of WT and Dsk7 mice
