# Supplementary material for: Gα11 mutation in mice causes hypocalcemia rectifiable by calcilytic therapy
Source: JCI Insight. 2017 Feb 9;2(3):e91103. doi: 10.1172/jci.insight.91103 (PMC5291742; doi:10.1172/jci.insight.91103)
Supplement: Supplemental data [file jciinsight-2-91103-s001.pdf]

Supplementary Table 1. Proportion of offspring bred from crosses of *Dsk7/+* x *Dsk7/+* mice

Genotype	Expected number of offspring (n=584 born)	Observed number of offspring (n=482 weaned)
+/+	146 (25%)	128 (27%)
Dsk7/+	292 (50%)	272 (56%)
Dsk7/Dsk7	146 (25%)	82 (17%)

Chi-square analysis showed the observed proportion of offspring to be different from those expected ($x^2 = 10.20$, $df = 2$, $P < 0.01$), with significantly fewer *Dsk7/Dsk7* mice being bred.

Supplementary Figure 1

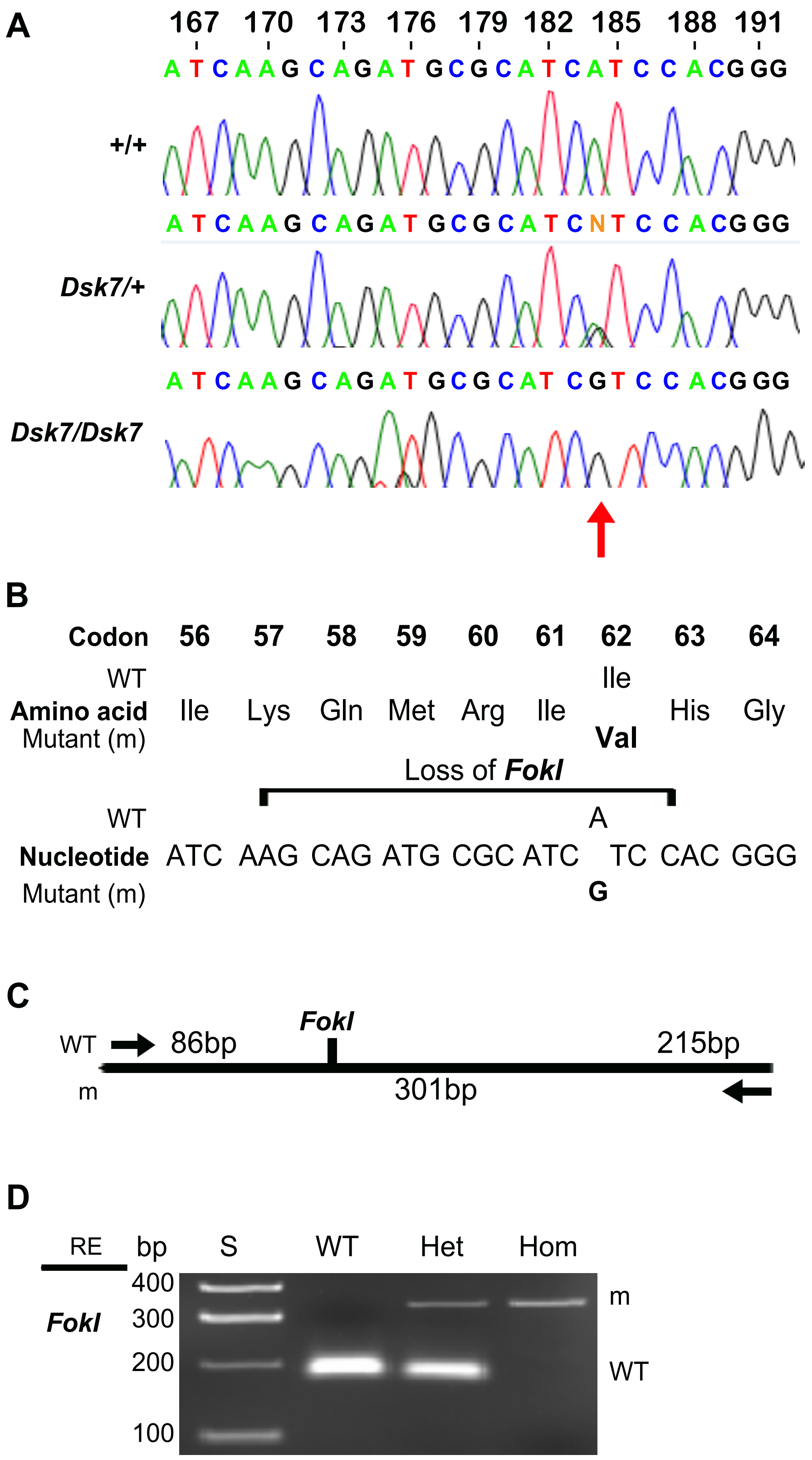

Supplementary Figure 1. DNA sequence and restriction endonuclease analysis of the Val62 Gα11 mutation. (**A**) DNA sequence analysis showed an A-to-G transition at c.184 (red arrow) within exon 2 of *Gna11*. (**B**) This sequence abnormality was predicted to lead to a missense substitution of Ile to Val at codon 62, resulting in the loss of a FokI restriction endonuclease (RE) site. (**C**) Restriction maps showing FokI digest would result in two products of 66bp and 160bp for the WT, and one 226bp product for the mutant (m). (**D**) RE digest of Gna11 exon 2 PCR products demonstrating the A-to-G transition in Dsk7/+ and Dsk7/Dsk7 mice. S = size marker.

Supplementary Figure 2

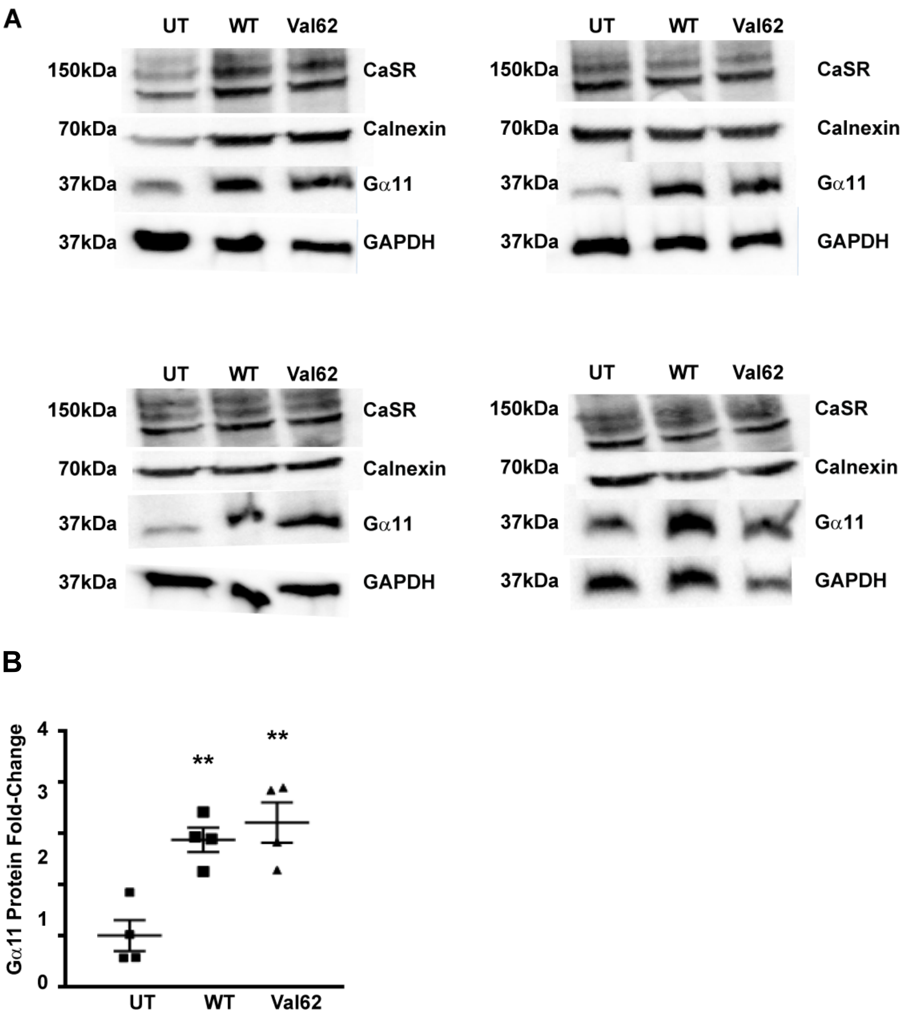

Supplementary Figure 2. Cells transiently transfected with WT and mutant Val62 pBI-CMV2-*GNA11* constructs overexpress the Ga$_{11}$ protein. (A) Four separate Western blots of lysates from HEK-CaSR cells transiently expressing WT (Ile62) or mutant (Val62) pBI-CMV2-*GNA11* constructs. Transient transfection with WT or mutant Val62 expression constructs resulted in overexpression of Gα$_{11}$ compared to untransfected cells (UT). GAPDH was used as a housekeeping protein for Gα$_{11}$ blots, and calnexin for CaSR blots. (B) Densitometric analysis of Gα$_{11}$ protein levels in each of the blots. Gα$_{11}$ expression was normalized to levels of GAPDH and expressed as a fold-change of Gα$_{11}$ expression in UT cells. Gα$_{11}$ was significantly overexpressed in transfected cells compared to UT cells. The expression of mutant Gα$_{11}$ with endogenous WT Gα$_{11}$ in transfected cells corresponded to the heterozygous situation *in vivo*. Data is expressed as mean±SEM of the 4 blots which represent 4 independent lysates. Statistical analyses were performed using two-way ANOVA. **P<0.01.

Supplementary Figure 3

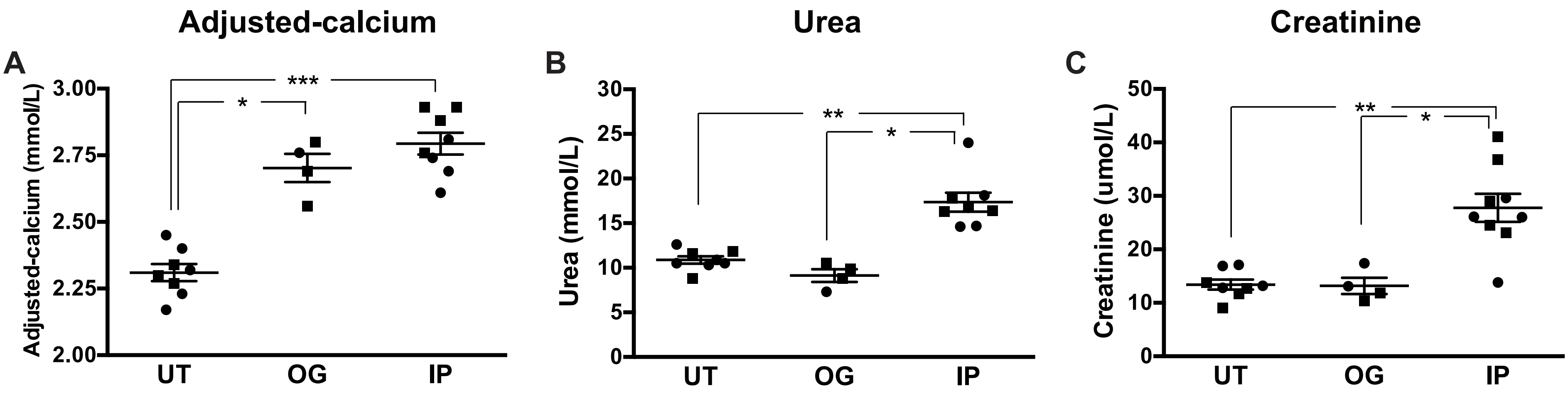

Supplementary Figure 3. Comparsion of route of administration of NPS-2143 on plasma calcium and renal function in WT mice. (**A**) plasma adjusted-calcium, (**B**) plasma urea, and (**C**) plasma creatinine concentrations of untreated (UT) WT mice, and of WT mice given NPS-2143 by oral gavage (OG) or intraperitoneal (IP) injection. Plasma biochemistry was measured at 1hr post-dose in NPS-2143-treated mice. Mean±SEM values for the respective groups are indicated by solid bars. Squares, males; circles, females. *P < 0·05,**P < 0·01, ***P < 0.001. A Kruskal-Wallis test followed by Dunn's test for non-parametric pairwise multiple comparisons were used for analysis of A-C.

Supplementary Figure 4

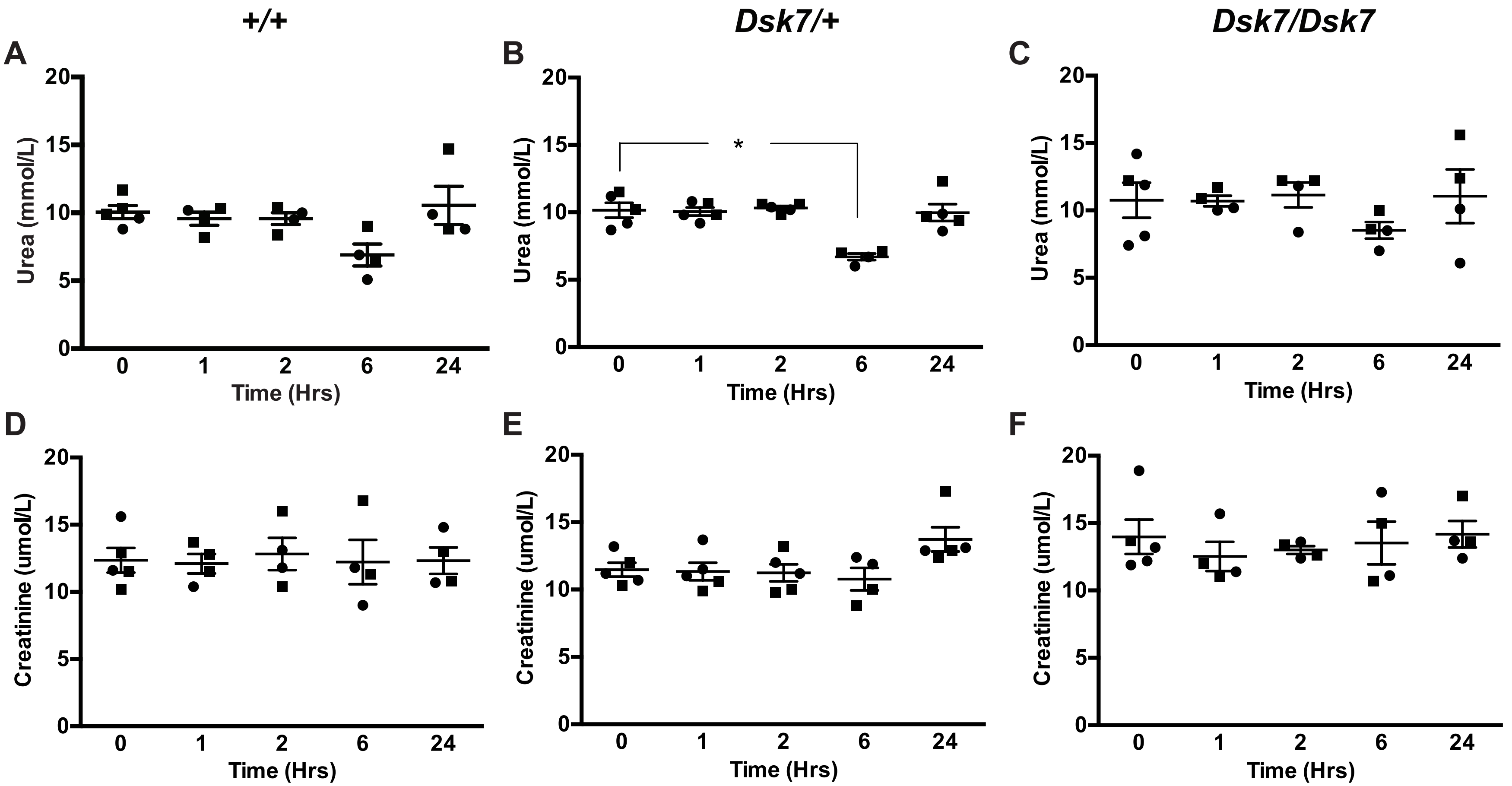

Supplementary Figure 4. Effect of NPS-2143 on plasma urea and creatinine of WT and Dsk7 mice at 0, 1, 2, 6 and 24hrs post-dose. (**A-C**) plasma urea and (**D-F**) plasma creatinine concentrations of WT (+/+), Dsk7/+ and Dsk7/Dsk7 mice, respectively. Mean±SEM values for the respective groups are indicated by solid bars. N=4-5 mice per study time-point. Squares, males; circles, females. *P < 0.05. A Kruskal-Wallis test followed by Dunn's test for non-parametric pairwise multiple comparisons were used for analysis of A-F.

Supplementary Figure 5

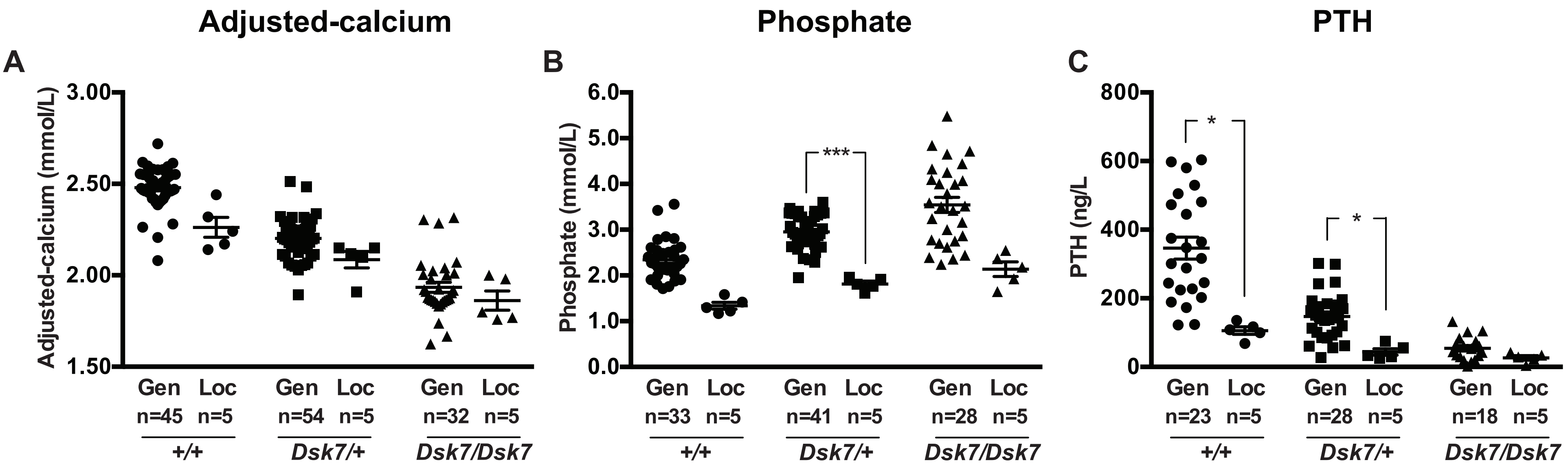

Supplementary Figure 5. Comparsion of effect of isoflurane general anesthesia versus topical local anesthesia on plasma concentrations of calcium, phosphate and PTH. (**A**) plasma adjusted-calcium, (**B**) plasma phosphate, and (**C**) plasma PTH concentrations of +/+ (WT), Dsk7/+ and Dsk7/Dsk7 mice. Mean±SEM values for the respective groups are indicated by solid bars. Circles, +/+ mice; squares, Dsk7/+ mice; triangles, Dsk7/Dsk7 mice. Gen, general isoflurane anesthesia; Loc, local anesthesia.*P < 0·05, ***P < 0.001. A Kruskal-Wallis test followed by Dunn's test for non-parametric pairwise multiple comparisons were used for analysis of A-C.
